# *Yersinia pestis* DNA Sequences in Late Medieval Skeletal Finds, Bavaria

**DOI:** 10.3201/eid1705.101777

**Published:** 2011-05

**Authors:** Thi-Nguyen-Ny Tran, Didier Raoult, Michel Drancourt

**Affiliations:** Author affiliation: Université de la Méditerranée, Marseille, France

**Keywords:** plague, Yersinia pestis, Black Death, genotyping, homopolymeric tract, bacteria, letter

**To the Editor:** We read with interest the report by Wiechmann et al. that, in the investigation of late medieval plague, partial sequencing of the *Yersinia pestis* pPCP1 plasmid yielded the observation of a 3-T homopolymeric tract which differed from the 5-T homopolymeric tract of the Orientalis *Y. pestis* CO92 type strain (*1*). This observation was unexpected because previous data from multispacer sequence typing and *glp* D gene sequencing yielded only the Orientalis biotype in cases of ancient plague (*2*).

Using suicide PCR (*3*), we therefore further investigated pPCP1 in 10 negative control dental pulp specimens and 60 specimens collected from 1 Justinian Orientalis plague site (*2*), 2 Black Death Orientalis sites, and 2 additional medieval plague sites. All negative controls remained negative; 14 (23%) of 60 plague specimens yielded a PCR product, and 7 interpretable sequences yielded a 3-T homopolymeric tract in all cases.

We further tested a *Y. pestis* isolate collection comprising 2 Antiqua, 6 Medievalis, and 4 Orientalis strains. No amplification was obtained in DNA-free PCR mix and 5 *Y. enterocolitica*–negative control isolates, whereas sequencing yielded a 3-T homopolymeric tract in all 12 *Y. pestis* isolates.

BLAST analysis (http://blast.ncbi.nlm.nih.gov/blast.cgi) indicated that the 5-T homopolymeric tract has been found only once in the *Y. pestis* CO92 strain (*4*) and in none of 22 modern and 11 ancient sequences (Table). This 5-T homopolymeric tract is therefore CO92 strain specific and not a marker for the Orientalis biotype. This pPCP1 plasmid sequence, located into a noncoding region of the 3′ extremity of the plasmid, is characterized by several homopolymeric tracts of poly (A) and poly (T), including the 1 herein investigated. Instability of the T-stretches has been reported in bacterial genomes (*5*) as being hot spots for mutations (*5*).

**Table:** Alignment of pPCP1 *Yersinis pestis* modern and ancient sequences

Source and *Y. pestis* strain	GenBank accession no.	Sequence, 5′ → 3′
Complete sequence		
*Y. pestis* CO92 plasmid pPCP1	AL109969.1	8488_TATATTTTCAAGAAAAGCTGGCTATTTAACATAACGGCAATTTTTGTACGCACCACTGAA_8547
*Y. pestis* KIM plasmid pPCP1	AF053945.1	8488_TATATTTTCAAGAAAAGCTGGCTATTTAACATAACGGCAATTT..GTACGCACCACTGAAAT_8547
*Y. pestis* biovar Microtus str. 91001 plasmid pPCP1	AE017046.1	8487_TATATTTTCAAGAAAAGCTGGCTATTTAACATAACGGCAATTT..GTACGCACCACTGAAAT_8546
*Y. pestis* Nepal516 plasmid pPCP	CP000307.1	9650_TATATTTTCAAGAAAAGCTGGCTATTTAACATAACGGCAATTT..GTACGCACCACTGAAAT_9709
*Y. pestis* Antiqua plasmid pPCP	CP000310.1	9661_TATATTTTCAAGAAAAGCTGGCTATTTAACATAACGGCAATTT..GTACGCACCACTGAAAT_9720
*Y. pestis* D182038 plasmid pPCP1	CP001592.1	8486_TATATTTTCAAGAAAAGCTGGCTATTTAACATAACGGCAATTT..GTACGCACCACTGAAAT_8545
*Y. pestis* Z176003 plasmid pPCP1	CP001596.1	8487_TATATTTTCAAGAAAAGCTGGCTATTTAACATAACGGCAATTT..GTACGCACCACTGAAAT_8546
Modern isolate		
103813 *Y. pestis* Nairobi rattus Antiqua	HQ542863	61_TATATTTTCAAGAAAAGCTGGCTATTTAACATAACGGCAATTT..GTACGCACCACTGAAAT_120
103814 *Y. pestis* JHUPRI Antiqua	HQ542864	61_TATATTTTCAAGAAAAGCTGGCTATTTAACATAACGGCAATTT..GTACGCACCACTGAAAT_120
103815 *Y. pestis* 14–47 Medievalis	HQ542865	61_TATATTTTCAAGAAAAGCTGGCTATTTAACATAACGGCAATTT..GTACGCACCACTGAAAT_120
103817 *Y. pestis* 5G5 Medievalis	HQ542866	61_TATATTTTCAAGAAAAGCTGGCTATTTAACATAACGGCAATTT..GTACGCACCACTGAAAT_120
103818 *Y. pestis* 5F1 Medievalis	HQ542867	61_TATATTTTCAAGAAAAGCTGGCTATTTAACATAACGGCAATTT..GTACGCACCACTGAAAT_120
103819 *Y. pestis* 6B4 Medievalis	HQ542868	61_TATATTTTCAAGAAAAGCTGGCTATTTAACATAACGGCAATTT..GTACGCACCACTGAAAT_120
103820 *Y. pestis* 8B7 Medievalis	HQ542869	61_TATATTTTCAAGAAAAGCTGGCTATTTAACATAACGGCAATTT..GTACGCACCACTGAAAT_120
103821 *Y. pestis* 9F11 Medievalis	HQ542870	61_TATATTTTCAAGAAAAGCTGGCTATTTAACATAACGGCAATTT..GTACGCACCACTGAAAT_120
103822 *Y. pestis* 6/69M Orientalis	HQ542871	61_TATATTTTCAAGAAAAGCTGGCTATTTAACATAACGGCAATTT..GTACGCACCACTGAAAT_120
103823 *Y. pestis* EV-76 Orientalis	HQ542872	61_TATATTTTCAAGAAAAGCTGGCTATTTAACATAACGGCAATTT..GTACGCACCAC_114
103824 *Y. pestis* algeria 1 Orientalis	HQ542873	64_TATATTTTCAAGAAAAGCTGGCTATTTAACATAACGGCAATTT..GTACGCACCACTGAAA_123
103825 *Y. pestis* algeria 2 Orientalis	HQ542874	61_TATATTTTCAAGAAAAGCTGGCTATTTAACATAACGGCAATTT..GTACGCACCACTGAAAT_120
Ancient strain detected from teeth		
Tooth no. 107 (excavated from Lariey site, France, 17th century)	HQ542875	62_TATATTTTCAAGAAAAGCTGGCTATTTAACATAACGGCAATTT..GTACGCACCACTGAAATGC_123
Tooth no. 515 (excavated from Venice site, Italy, 14th–16th centuries)	HQ542876	60_TATATTTTCAAGAAAAGCTGGCTATTTAACATAACGGCAATTT..GTACGCACCACTGAAATGC_121
Tooth no.1183 (excavated from Bondy site, France, 11th–15th centuries)	HQ542877	62_TATATTTTCAAGAAAAGCTGGCTATTTAACATAACGGCAATTT..GTACGCACCACTGAAAT_121
Tooth no. 1184 (excavated from Bondy site, France, 11th–15th centuries)	HQ542878	61_TATATTTTCAAGAAAAGCTGGCTATTTAACATAACGGCAATTT..GTACGCACCACTGAAAT_121
Tooth no. 1190 (excavated from Bondy site, France, 11th–15th centuries)	HQ542879	61_TATATTTTCAAGAAAAGCTGGCTATTTAACATAACGGCAATTT..GTACGCACCACTGAAAT_120
Tooth no. 254 (excavated from Venice site, Italy, 14th–16th centuries)	HQ542880	61_TATATTTTCAAGAAAAGCTGGCTATTTAACATAACGGCAATTT..GTACGCACCACTGAAAT_120
Tooth no. 1180 (excavated from Bondy site, France, 11th–15th centuries)	HQ542881	61_TATATTTTCAAGAAAAGCTGGCTATTTAACATAACGGCAATTT..GTACGCACCACTGAAAT_120

Therefore, in our assessment, the data reported for the late medieval Bavaria burial (*1*) do not support that deaths of persons buried in this site resulted from a non-Orientalis plague. Typing modern or ancient *Y. pestis* strains should not rely on poly (A) and poly (T) homopolymeric tracts sequencing.
