# Supplementary material for: Yersinia pestis DNA Sequences in Late Medieval Skeletal Finds, Bavaria
Source: Emerg Infect Dis. 2011 May;17(5):955–7. doi: 10.3201/eid1705.101777 (PMC3338162; doi:10.3201/eid1705.101777)
Supplement: Related texts

# Reply: *Yersinia pestis* DNA Sequences in Late Medieval Skeletal Finds, Bavaria

**Authors:** Ingrid Wiechmann, Michaela Harbeck, Gisela Grupe

**Affiliations:** Author affiliations: Ludwig Maximilian University of Munich, Munich, Germany (I. Wiechmann, G. Grupe); Bavarian State Collection of Anthropology and Palaeoanatomy, Munich (M. Harbeck, G. Grupe)

**In Response:** We thank Tran et al. for their interest in our article (*1*). In it, we described the detection of several *Yersinia pestis*–specific plasmid pPCP1 DNA sequences in skeletal remains from a late medieval mass burial in Bavaria, Germany. In 1 of these sequence sections, we found a deviation from the reference sequence used (*Y. pestis* strain CO92 plasmid sequence AL109969.1). We did not further interpret this result because we agree with Tran et al. that typing of *Y. pestis* strains should not rely on poly (A) and poly (T) homopolymeric tract sequencing (*2*). As we have stated (*1*), further analyses of our material, including chromosomal markers (*3**,**4*) will be conducted to obtain clues as to the specific *Y. pestis* strain.

## References

[1] Wiechmann I, Harbeck M, Grupe G. *Yersinia pestis* DNA sequences in late medieval skeletal finds, Bavaria [letter]. Emerg Infect Dis. 2010;16:1806–7.2102955510.3201/eid1611.100598PMC3294523

[2] Tran T-N-N, Raoult D, Drancourt D. *Yersinia pestis* DNA sequences in late medieval skeletal finds, Bavaria [letter]. Emerg Infect Dis. 2011;17:955–7.2152943110.3201/eid1705.101777PMC3338162

[3] Morelli G, Song Y, Mazzoni CJ, Eppinger M, Roumagnac P, Wagner DM, *Yersinia pestis* genome sequencing identifies patterns of global phylogenetic diversity. Nat Genet. 2010;42:1140–3. 10.1038/ng.70521037571PMC2999892

[4] Haensch S, Bianucci R, Signoli M, Rajerison M, Schultz M, Kacki S, Distinct clones of *Yersinia pestis* caused the Black Death. PLoS Pathog. 2010;6:e1001134. 10.1371/journal.ppat.100113420949072PMC2951374
